# Positive regulatory effects of perioperative probiotic treatment on postoperative liver complications after colorectal liver metastases surgery: a double-center and double-blind randomized clinical trial

**DOI:** 10.1186/s12876-015-0260-z

**Published:** 2015-03-20

**Authors:** Zhihua Liu, Chao Li, Meijin Huang, Chao Tong, Xingwei Zhang, Lei Wang, Hui Peng, Ping Lan, Peng Zhang, Nanqi Huang, Junsheng Peng, Xiaojian Wu, Yanxing Luo, Huanlong Qin, Liang Kang, Jianping Wang

**Affiliations:** 1 Gastrointestinal Institute of Sun Yat-sen University, Department of Colorectal Surgery, the Sixth Affiliated Hospital of Sun Yat-sen University (Guangdong Gastrointestinal Hospital), 26 Yuancun Erheng Road, Guangzhou, Guangdong 510655 People’s Republic of China; 2 Department of Surgery, Shanghai JiaoTong University Affiliated Sixth People’s Hospital, Shanghai, 200233 China

**Keywords:** Probiotics, Colorectal liver metastases, Zonulin, Postoperative complication, Liver barrier

## Abstract

**Background:**

Colorectal liver metastases (CLM) occur frequently and postoperative intestinal infection is a common complication. Our previous study showed that probiotics could decrease the rate of infectious complications after colectomy for colorectal cancer. To determine the effects of the perioperative administration of probiotics on serum zonulin levels which is a marker of intestinal permeability and the subsequent impact on postoperative infectious complications in patients with CLM.

**Methods:**

150 patients with CLM were randomly divided into control group (n = 68) and probiotics group (n = 66). Probiotics and placebo were given orally for 6 days preoperatively and 10 days postoperatively to control group and probiotics group respectively. We used the local resection for metastatic tumor ,while for large tumor, the segmental hepatectomy. Postoperative outcome were recorded. Furthermore, complications in patients with normal intestinal barrier function and the relation with serum zonulin were analyzed to evaluate the impact on the liver barrier dysfunction.

**Results:**

The incidence of infectious complications in the probiotics group was lower than control group. Analysis of CLM patients with normal postoperative intestinal barrier function paralleled with the serum zonulin level. And probiotics could also reduce the concentration of serum zonulin (P = 0.004) and plasma endotoxin (P < 0.001).

**Conclusion:**

Perioperative probiotics treatment could reduce the serum zonulin level, the rate of postoperative septicemia and maintain the liver barrier in patients undergoing CLM surgery. we propose a new model about the regulation of probiotics to liver barrier via clinical regulatory pathway. We recommend the preoperative oral intake of probiotics combined with postoperative continued probiotics treatment in patients who undergo CLM surgery.

**Trial registration:**

ChiCTR-TRC-12002841. 2012/12/21

**Electronic supplementary material:**

The online version of this article (doi:10.1186/s12876-015-0260-z) contains supplementary material, which is available to authorized users.

## Background

The liver is a common site for metastatic disease [1]. Over 50% of the primary tumor are originated from the gastrointestinal tract, especially colon and rectum [2]. Because morbidity and mortality of colorectal cancer (CRC) increased year by year, CRC has been ranked as the second cause of tumor death around the world [3] and liver is the most common site of metastases of CRC [4-6]. Resection of colorectal liver metastases (CLM) is the best choice for the treatment of CRC with liver metastasis [7], it is reported that simultaneous resection is as efficient as a delayed procedure for long-term survival [8].

With the development of advanced surgical techniques, postoperative survival rate of CLM has been considerably promoted [9]. However, the incidence of postoperative infectious complications are becoming more and more common [10,11]. These complications may also lead to higher rates of recurrence and death [12-15].

It is known to all that PRO play an important role in the stability of the intestinal microbiological environment [16,17]. In recent years, some randomized clinical trials using PRO for to surgical patients for the protective effects perioperatively [18-20] could regulate intestinal microbial populations, lower intestinal permeability, decrease the incidence of infection-related complications and overcome other problems, significantly.

Zonulin, the only known physiological modulator of intercellular TJ described so far, is a protein which constitutes tight junctions of the digestive tract and modulates intestinal permeability [1,21]. Recently, zonulin was found to be a reflection of the intestinal permeability [22]. It is reported that elevated levels of plasma zonulin in septic patients might serve as a mechanism for increased intestinal permeability in sepsis and systemic inflammatory response syndrome (SIRS) [23]. Another study showed that zonulin both in vitro and in vivo induced generation of complement C3a and C5a, suggesting that zonulin facilitated the development of acute lung injury and lung barrier by enhancing albumin leak and complement activation as well as increased build up of neutrophils and cytokines [24]. However, no study has explored the link about zonulin, liver barrier and the rates of complications after CLM surgery, the specific role of PRO has not been elucidated yet.

Our previous study indicated that PRO could increase transepithelial resistance (TER) and increase fecal bacterial variety, reduce transmucosal permeability of horseradish peroxidase, lactulose/mannitol ratio, bacterial translocation rate, ileal-bile acid binding protein, positive rate of blood bacterial DNA, blood enteropathogenic bacteria [11]. Further study showed that PRO decreased the serum zonulin concentration, the duration of postoperative pyrexia, duration of antibiotic therapy and the rate of postoperative infective complications in patients underwent colectomy [1].

As liver barrier may have an impact on the incidence of postoperative liver complications, we proposed the hypothesis that pre- and postoperative PRO may reduce liver permeability, decrease the rate of bacterial translocation (BT) and infectious complications after CLM surgery. we aimed to investigate the effects of the perioperative administration of PRO on serum zonulin levels, liver permeability and the subsequent impact on postoperative liver complications in patients undergoing CLM.

## Methods

### Inclusion and exclusion criteria

Inclusion criteria in our study included: 1) age between 25 and 75 years; 2) the diagnosis of CRC were confirmed by biopsy and histological testing, liver metastases were diagnosed by CT preoperatively and confirmed by postoperative histological report; 3) patients underwent a radical resection of primary colorectal tumors and liver metastases; and 4) they had no other metastasis.

The exclusion criteria were as follows: 1) pregnant; 3) lactose intolerance; 4) clinically significant immunodeficiency; 5) gastrointestinal disorders (e.g., Crohn’s disease or ulcerative colitis); 6) received antibiotics during the 10 days before surgery; 7) infection; 8) probiotic or excessive fiber intake within 2 weeks of surgery; 9)underwent an emergency operation; 10) bowel preparation for colonoscopy prior to surgery within 6 days; 11)underwent a proctectomy with low rectal anastomosis or surgery for a polypoid lesion; 12) the surgery was performed laparoscopically; 13) received preoperative neoadjuvant chemotherapy or radiotherapy; 14) had other distant metastases except liver; 15) unresectable liver metastases; and 16) severe liver function failure or other organ failure.

### Patients

150 patients with CLM were scheduled to undergo colectomy at the Shanghai Sixth People’s Hospital, affiliated to Shanghai JiaoTong University in Shanghai or the Sixth Affiliated Hospital of Sun Yat-sen University in Guangzhou, between April 2007 and July 2013. The patients were randomized prior to surgery to the placebo control group (control) with perioperative oral feeding placebo or the PRO therapy group (PRO) with PRO treatment pre- and post-operatively. The study protocols were reviewed and approved by the Human Research Review Committee of the two hospitals and written informed consent for participation were obtained from each patient [1]. The requirement for informed consent was waived by each of the institutional review boards (IRBs) that approved the study (Human Research Review Committee of the The Sixth Affiliated Hospital of Sun Yat-sen University).

**Figure 1:**
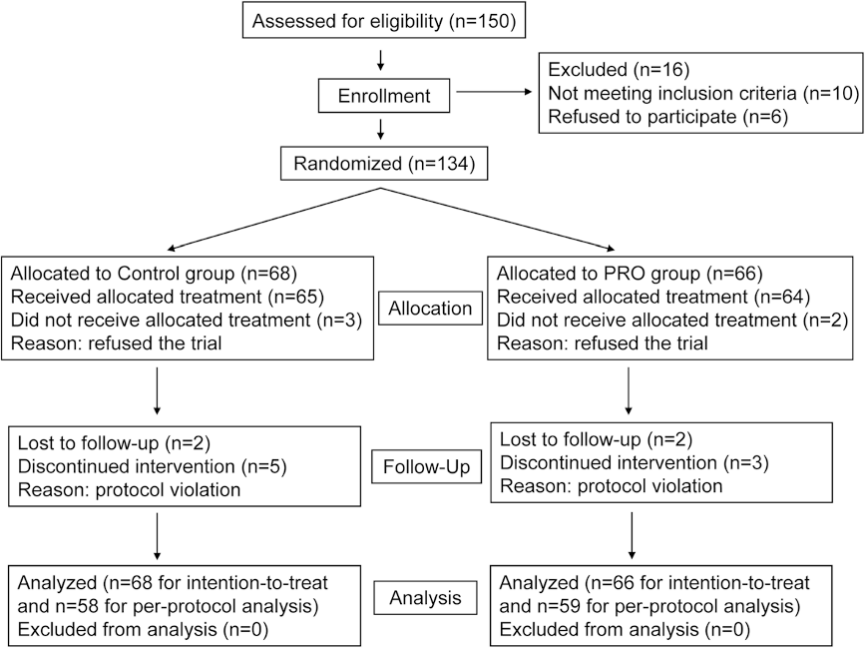
**Flow chart of the randomization procedure used to enroll patients in the study.**

All patients were assessed for eligibility, while 16 patients were excluded, for whom did not meet the inclusion criteria (10 patients) or refused to participate (6 patients). 117 of the 134 patients completed the entire study (Figure 1). There were no significant differences in sex, age, BMI, time between the onset of symptoms and hospital admission, no significant difference was found in the preoperative serum levels of albumin, Hb, creatinine, and operative time, intra-operative blood loss, intra-operative transfusion, usage of supplemental albumin postoperation, preoperative preparation time the number of patients treated with metronidazole, penicillin, ceftriaxone, liver function (ALT and AST) (Table 1 for intention-to-treat and Additional file 1: Table S1 for per-protocol).

**Table 1:** **Baseline of characteristics of the patients with colorectal liver metastases undergoing surgery at hospital admission in the study (Intention-to-treat)**

Index	Control group (n = 68)	PRO group (n = 66)
Sex (Male/Female)	35/33	35/31
Age (Year)	60.16 ± 16.20	65.62 ± 18.18
BMI (kg/m^2^)	23.06 ± 5.26	22.28 ± 3.66
Location of tumor		
ascending colon	15	15
Transverse colon	6	5
Descending colon	12	16
Sigmoid colon	15	11
Rectum	20	19
Time between onset of symptoms and hospital admission (d)	50.28 ± 16.62	55.12 ± 18.26
Preoperative albumin (g/dL)	38.26 ± 8.56	36.98 ± 6.96
Preoperative Hb (g/L)	126.86 ± 32.06	116.22 ± 36.68
Creatinine (mg/dL)	1.26 ± 0.28	1.20 ± 0.62
Operative time (min)	186.28 ± 56.36	199.82 ± 55.98
Intra-operative blood loss (ml)	336.26 ± 182.68	352.56 ± 169.26
Transfusion during operation (ml)	308.12 ± 120.66	329.16 ± 130.58
Usage of supplemental albumin postoperation (g)	22.38 ± 16.36	28.12 ± 20.98
Preoperation prepared time (d)	6.12 ± 3.52	6.68 ± 2.98
Metronidazole (n)	68	66
Penicillin (n)	30	33
Ceftriaxone (n)	38	33
ALT (U/L)	35.68 ± 15.26	32.62 ± 18.86
AST (U/L)	29.68 ± 16.56	28.22 ± 18.86

BMI, body mass index; Hb, hemoglobin; ALT, alanine transarninase (normal value, 0–40 U/L); AST, aspartate aminotransferase (normal value, 0–40 U/L).

There were no significant differences about the characteristics between the two groups.

Quantitative data are expressed as mean ± standard deviation. Numerical data were compared by t test and nominal data by Pearson χ^2^ test or Fisher’s exact test between groups.

### Study design, PRO treatment, and patient care

Randomization to two groups was accomplished using double randomization principle (with the help of envelopes and random variation row) constitution in our patient department of the Sixth Affiliated Hospital of Sun Yat-sen University. Only a nurse knew the treatment assignment who was not directly involved in the trial and could broke the treatment codes in the event of an emergency.

Patients in the PRO group received encapsulated admixture of three PRO bacteria (Institute of Life Science of Only, Shanghai Jiao Tong University, Shanghai, China), composed of LP (CGMCC No.1258, cell count ≥10^11^ cfu/g), LA-11 (cell count ≥7.0 × 10^10^ cfu/g) and BL-88 (cell count ≥5.0 × 10^10^ cfu/g) every day. An acid-resistant coating was used to prepare the capsules which wrapping the PRO a or placebo. Each patient in the PRO group received PRO 2 g/day, at a total daily dose of 2.6 × 10^14^ cfu. Patients in the control group received daily encapsulated maltodextrin. The appearance, smell and taste of the two types of capsules showed no obvious difference.

The intervention period lasted 16 days, 6 days preoperatively and 10 days postoperatively. All the subjects were interviewed by the study nurse, any reactions to the product, medications taken and adverse events that occurred were recorded.

During the study period, no parenteral or enteral nutritional supplementation was given. All patients received a regular diet preoperatively, and a low-residue diet 1 day preoperatively. Mechanical bowel preparation was given 1 day before the surgery, in which all patients were given Soffodex, containing 2.4 g of monobasic sodium phosphate and 0.9 g of dibasic sodium phosphate. Parenteral hydration was given on the morning of the surgery via a central venous catheter. A catheter was placed for gastric aspiration to reduce flow through the colon anastomosis. 500 mg of metronidazole and 1 g of ceftriaxone were given 1 h before induction and continued for 48 h after surgery. After the surgery, all patients received regular parenteral hydration. Complications were registered daily and patients were re-examined at the outpatient clinic 1, 2, and 4 weeks after surgery.

### Postoperative clinical observations

Detailed postoperative records were kept daily and infectious complications were recorded for up to 30 days after surgery. The diagnosis of bacterial infection was based on the previous reference [11,19] and several types of complications were observed after surgery. The septicemia incidence, liver function after 10 days postoperative, the use of PRO, intra-abdominal drainage time and infections-related complication were recorded. One of the three doctors examine the patients (Liang Kang, Peng Zhang, and Chao Li) to evaluate bowel sounds, abdominal cramping, and distension on the postoperative days.

Our previous study [1] indicated that PRO lower the intestinal permeability and the postoperative septicemia rate. In this study, we further investigate the effects of PRO on the liver barrier by comparing the postoperative infection-related complications with normal intestinal permeability at day 3 after surgery to eliminate the interference of intestinal barrier.

### Measurement of serum zonulin concentration

The concentration of serum zonulin collected at 24 h postoperatively were determined using an ELISA kit [25]. Briefly, plastic microtiter plates (Costar, Cambridge, MA) were coated with rabbit zonulin cross-reacting Zot derivative ΔG IgG antibodies (10 μg/ml in 0.1 mol/l sodium carbonate buffer, pH 9.0) [25]. After an overnight incubation at 4°C, plates were washed four times in TBS-T and blocked by incubation for 1 h at 37°C with TBS-T. After four washes in TBS-T, five ΔG serial standards (50, 25, 12.5, 6.2, 3.1, and 0 ng/ml) and patient sera samples (1:101 dilution in TBS-T) were added and incubated overnight at 4°C. After four washes with Tris-buffered saline 0.2% Tween 20 buffer, plates were incubated with biotinylated anti-Zot IgG antibodies for 4 h at 4°C. A color reaction was developed using a commercial kit (ELISA amplification kit; Invitrogen). The absorbance at 495 nm was measured with a microplate auto-reader (Molecular Devices Thermomax Microplate Reader).

### Intestinal permeability assay

Intestinal permeability was assessed using the L/M test preoperatively (the morning of the surgery) and on the 3^th^ and 10^th^ day postoperatively [1,11]. After an overnight fast, all subjects were given the oral test solution containing 10 g of lactulose (Sigma-Aldrich, Tokyo, Japan) and 5 g of mannitol (Sigma-Aldrich) in 60 ml of physiological saline. For the next 6 h, the subjects were rested and no food or water was allowed. Complete 6 h urine collections were taken and a further 10-mL urine sample was frozen at −20°C until analysis. Urinary lactulose and mannitol concentrations were measured by gas–liquid chromatography.

### Measurement of plasma concentrations of endotoxin

Plasma samples used for endotoxin measurements were stored in endotoxin-free glass tubes to prevent the loss of endotoxin to plastic tubes walls. All materials used for the assay were rendered endotoxin-free. Plasma endotoxin concentrations were measured by a commercially available kit [Cambrex Limulus Amebocyte Lysate (LAL) kit; Lonza Inc, Walkersville, MD] [26]. LPS from the sample reacts enzymatically with a proenzyme in the LAL reagent that leads to its activation and the production of a colored peptide from the chromogenic substrate reagent over a short incubation period that can be read at 405–410 nm.

### Microbiological investigations and PCR assay for bacterial DNA fragment

Clinical samples comprising blood (40 mL), central lines (tips), urine (20 mL), and sputum were collected at 06:00 [11]. Each clinical sample was taken approximately 72 h after the operation, and immediately sent to the microbiological laboratory. The specimens were cultured under aerobic condition, microaerophilic condition and anaerobic condition respectively at 35-37°C for 24–48 h. Anaerobic cultivation was performed in anaerobic chamber. Sabouraud’s medium (bioMérieux, France) were used in the Fungal cultures. The biochemical characteristics of the cultured strains were investigated using the API and/or ID tests (bioMérieux, France). The remaining 20 mL of blood was collected in a sterile container containing EDTA. To determine the sensitivity of the PCR detection, serial dilutions of the spiked blood were tested until a negative result. The sensitivity of the test was 10 organisms/mL.

### RNA extraction, cDNA synthesis, and real-time quantitative PCR (qRT-PCR)

Our previous study indicated that the p38 mitogen-activated protein kinase (MAPK) signaling pathway was involved in the protective process of intestinal permeability and intestinal barrier function [27]. Thus, we determined the liver p38 MAPK gene expression using the RT-PCR [25,27]. Briefly, total RNA was extracted from the adjacent normal liver tissues of five patients with CLM using Trizol reagent (Invitrogen, Grand Island, NY) to detect the expression level of p38 MAPK. For each sample, 600 ng of mRNA was used in the reverse transcription reaction (iScript kit from BioRad Laboratories, Hercules, CA, USA). Further analysis of the mRNA levels of each group was performed by real time PCR with a light-cycling system (LightCycler; Roche Diagnostics GmbH, Mannheim, Germany). All values were expressed as a fold increase or decrease compare with the expression of actin. The sequences of the primers were as follows: p38 MAPK, F: 5′-GAAGAGCCTGACCTACGAT-3′ and R: 5′-ACTGCCAAGGAGCATCTA-3′; β-actin, F: 5′-CTCCATCCTGGCCTCGCTGT-3′ and R: 5′-GCTGTCACCTTCACCGTTCC-3′.

### Statistical analysis

A sample size calculation based on the prevalence of BT demonstrated that approximately 120 patients would be required in each group to demonstrate a reduction in BT from 25% to 5% at the 5% significance level with a power of 80% [1]. Results were analyzed using SPSS 13.0 version for Windows (SPSS, Chicago, Illinois, USA). Quantitative data are expressed as mean ± standard deviation. For postoperative clinical tests, numerical data were compared by t-tests and nominal data were analyzed between groups with the Pearson χ^2^ test or Fisher’s exact test. A P-value <0.05 was considered statistically significant. Spearman’s correlation was used to assess the relationship between zonulin level and outcome using SPSS 13.0.

## Results and discussion

### PRO reduced postoperative intestinal infection related complications and promoted rapid recovery

We first performed the comparison of overall postoperative outcomes between PRO and control groups (Table 2 & Additional file 2: Table S2), which verified our previous study [1]. Among the 134 patients, no death case was found, no patients got complications related leakage of the anastomosis, fistulas, and abdominal hemorrhage, no side effect of probiotic was reported in our study. Results indicated that the incidence of infectious complications in the PRO group was lower than the control group, such as septicemia incidence (59% vs 88%, P = 0.008), urinary infection (2% vs 13%, P = 0.017), diarrhea incidence (24% vs 46%, P = 0.012), then decrease the duration of postoperative pyrexia (6.02 ± 1.68 vs 6.98 ± 2.22, P = 0.006), cumulative duration of antibiotic therapy (6.22 ± 1.96 vs 7.56 ± 2.26, P < 0.001), postoperative hospital stay (11.26 ± 2.52 vs 12.96 ± 3.06, P < 0.001), and the overall hospital charge (52261.16 ± 12168.28 vs 58262.36 ± 10262.36, P = 0.002). For the liver function indexes, both ALT and AST were significantly lowered by the treatment of PRO (ALT, control vs PRO, 56.20 ± 18.16 vs 36.28 ± 18.92, P < 0.001; AST, control vs PRO, 45.62 ± 22.68 vs 36.18 ± 21.52, P = 0.015, Table 2). The gastrointestinal recover quicker in the PRO group for PRO group got shorter first defecation time (control vs PRO, 3.6 ± 1.8 vs 2.8 ± 1.6, P = 0.007), less abdominal cramping (control vs PRO, 49% vs 23%, P = 0.017), and abdominal distension (control vs PRO, 51% vs 33%, P = 0.038).

**Table 2:** **Comparison of postoperative outcomes between probiotics and control (Intention-to-treat)**

Outcomes	Intention-to-treat		
	Control (n = 68)	PRO (n = 66)	P Value
Septicemia incidence (%)	88 (60/68)	59 (39/66)	0.008
ALT (U/L)	56.20 ± 18.16	36.28 ± 18.92	<0.001
AST (U/L)	45.62 ± 22.68	36.18 ± 21.52	0.015
Intro-abdominal drainage time (d)	4.2 ± 1.6	4.6 ± 1.8	0.176
Incision infection (%)	12 (8/68)	9 (6/66)	0.779
Central lines infection (%)	9 (6/68)	11 (7/66)	0.777
Pneumonia infection (%)	12 (8/68)	9 (6/66)	0.097
Urinary infection (%)	13 (9/68)	2 (1/66)	0.017
First defecation time (d)	3.6 ± 1.8	2.8 ± 1.6	0.007
Diarrhea incidence (%)	46 (31/68)	24 (16/66)	0.012
Urinary catheters time (d)	7.1 ± 2.6	6.6 ± 2.8	0.286
Abdominal cramping (%)	49 (33/68)	23 (15/66)	0.017
Abdominal distension (%)	51 (35/68)	33 (22/66)	0.038
Intake time of fluid diet (d)	3.6 ± 1.2	3.2 ± 1.8	0.131
Intake time of solid diet (d)	5.2 ± 1.6	4.9 ± 1.6	0.280
Side effects of probiotic use	0	0	N/A
Duration of postoperative pyrexia (>38.5°C) (d)	6.98 ± 2.22	6.02 ± 1.68	0.006
Hypoalbuminemia (%)	47 (32/68)	27 (18/66)	0.021
Cumulative duration of antibiotic therapy	7.56 ± 2.26	6.22 ± 1.96	<0.001
Postoperative hospital stay	12.96 ± 3.06	11.26 ± 2.52	<0.001
Hospital charge (Yuan)	58262.36 ± 10262.36	52261.16 ± 12168.28	0.002
Death case	0	0	N/A
Serum zonulin (ng/mg protein)	1.36 ± 0.50	0.42 ± 0.36	<0.001

ALT, alanine transarninase (normal value, 0–40 U/L); AST, aspartate aminotransferase (normal value, 0–40 U/L).

### PRO reduce the postoperative serum zonulin concentration

The serum zonulin was not significantly different between the two groups (0.30 ± 0.62 ng/mg protein vs. 0.36 ± 0.38 ng/mg protein, P = 0.502) perioperately. The serum zonulin concentration in the control group (1.36 ± 0.50 ng/mg protein) was significantly higher than in the PRO group (0.42 ± 0.36 ng/mg protein, P < 0.001, Table 2) after 10 d postoperative treatment. Results indicated that PRO lower the postoperative zonulin level efficiently after CLM surgery. Ccompared with the results in our pervious study, which indicated that PRO could reduce the zonulin level after CRC surgery without liver metastases, the postoperative zonulin level of the control group was higher in our present study of CRC with liver metastases (CLM), compared with that without liver metastases (1.36 ± 0.50 ng/mg protein vs 1.08 ± 0.28 ng/mg protein). And postoperative septicemia incidence in the control group was also higher in our present study (88% vs 73%, P = 0.034). Therefore, we hypothesized that another barrier also played a role in the change of postoperative zonulin level inCLM, such as liver barrier. We plan to investigate the effects of PRO on the liver barrier.

We use the intestinal permeability to exclude patients with intestinal barrier dysfunction. Only patients with normal intestinal permeability at day 3 postoperatively, were included in the following analysis to eliminate the intestinal interference (Table 3 & Additional file 3: Table S3). We use the serum zonulin level before treatment to estimate the normal interval zonulin level. Intestinal permeability was assessed using the L/M test to distinguish the normal level of intestinal permeability from higher level at postoperative day 3. The average L/M ratio (including the ratio of control and PRO groups) before treatment was 0.161 ± 0.059, with the 95 confidence interval was (0.151, 0.171). Therefore, we only analyze the patients with the L/M ratio less than 0.171, which were confirmed 3 d after CLM surgery. Further analysis also indicated that there was no significant difference between the control and PRO groups before treatment (0.156 ± 0.062 vs 0.167 ± 0.056, P = 0.287).

**Table 3:** **Comparison of serum zonulin with the postoperative infectious complications between probiotics and control the patients with normal intestinal barrier function (Intention-to-treat)**

Outcomes	Intention-to-treat		
	Control (n = 30)	PRO (n = 30)	P Value
**Serum zonulin (ng/mg protein)**	0.72 ± 0.26	0.51 ± 0.29	0.004
**Septicemia (%)**			
Total	87 (26/30)	50 (15/30)	0.005
HZ	95 (19/20)	66 (10/15)	0.064
LZ	70 (7/10)	33 (5/15)	0.111
HZ vs. LZ	HZ vs LZ (83% vs 48%), P = 0.006		
Correlation between septicemia and zonulin, r = 0.613, P < 0.001			
**Urinary infection (%)**			
Total	20 (6/30)	0 (0/30)	0.024
HZ	15 (3/20)	0 (0/15)	0.244
LZ	30 (3/10)	0 (0/15)	0.052
HZ vs. LZ	HZ vs LZ (9% vs 12%), P = 0.686		
**Diarrhea incidence (%)**			
Total	60 (18/30)	27 (8/30)	0.018
HZ	50 (10/20)	27 (4/15)	0.296
LZ	80 (8/10)	27 (4/15)	0.015
HZ vs. LZ	HZ vs LZ (40% vs 48%), P = 0.603		
**Duration of postoperative pyrexia (>38.5°C) (d)**			
Total	6.99 ± 2.38	5.49 ± 3.21	0.044
HZ	7.97 ± 1.77 (n = 20)	8.02 ± 0.87 (n = 15)	0.927
LZ	5.01 ± 2.27 (n = 10)	2.95 ± 2.61 (n = 15)	0.054
HZ vs. LZ	HZ vs LZ (7.99 ± 1.43 vs 3.78 ± 2.64), P < 0.001		
**Cumulative duration of antibiotic therapy (d)**			
Total	7.17 ± 1.60	6.13 ± 1.72	0.019
HZ	7.90 ± 1.21 (n = 20)	7.47 ± 1.19 (n = 15)	0.298
LZ	5.70 ± 1.25 (n = 10)	4.80 ± 0.94 (n = 15)	0.052
HZ vs. LZ	HZ vs LZ (7.71 ± 1.20 vs 5.16 ± 1.14), P < 0.001		
**Postoperative hospital stay**			
Total	12.87 ± 3.01	11.33 ± 2.22	0.029
HZ	14.15 ± 2.64 (n = 20)	13.13 ± 1.60 (n = 15)	0.197
LZ	10.30 ± 1.89 (n = 10)	9.53 ± 0.83 (n = 15)	0.177
HZ vs. LZ	HZ vs LZ (13.71 ± 2.28 vs 9.84 ± 1.38), P < 0.001		
**Hospital charge (Yuan)**	60196.12 ± 6532.16	53628.22 ± 6513.28	<0.001

HZ, high serum zonulin (≥0.6 ng/mg protein); LZ, low serum zonulin (<0.6 ng/mg protein); total = HZ + LZ; NS, No significance.

Numerical data between groups were compared by the t-test and nominal data by Pearson χ^2^ test or Fisher’s exact test.

HZ vs LZ compares these subgroups without regard to treatment.

There was also a significant correlation between zonulin and duration of postoperative pyrexia (r = 0.919, p < 0.001), cumulative duration of antibiotic therapy and zonulin (r = 0.936, p < 0.001), and postoperative hospital stay (r = 0.911, p < 0.001).

### PRO lowered postoperative infection related complications in patients with normal intestinal barrier function

The baseline characteristics of patients with normal intestinal barrier function were re-evaluated. There was no significant difference about the baseline characteristics between the two groups (detailed in Additional file 4: Table S4). The postoperative infection-related parameters showed significant difference in Table 3 of intention-to-treat groups were selected for further analysis. For the analysis of patients with normal intestinal barrier function, serum zonulin level was not significantly different before treatment (0.32 ± 0.60 ng/mg protein vs. 0.36 ± 0.39 ng/mg protein, P = 0.761); while the serum zonulin concentration after 10 d postoperative treatment in the control group was significantly higher than in the PRO group (0.72 ± 0.26 ng/mg protein vs. 0.51 ± 0.29 ng/mg protein, P = 0.004, Table 3). The incidence of postoperative septicemia was 87% (26/30) in the control and 50% (15/30) in the PRO group (P = 0.005). Further analysis found that the incidence of postoperative septicemia with high serum zonulin levels did not differ between the two groups (95 (19/20) vs. 66 (10/15), P =0.064). . When patients were grouped according to serum zonulin level, the incidence of postoperative septicemia in the high serum zonulin group was significantly higher than that in the low serum zonulin group (83% (29/35) vs. 48% (12/25), P =0.006, Table 3). Similar results were indicated in the per-protocol analysis (Additional file 3: Table S3). The duration of postoperative pyrexia, the cumulative duration of antibiotic therapy and postoperative hospital stay all showed similar results to those of septicemia. The detailed comparison between the two groups is shown in Table 3 for the intention-to-treat analysis and Additional file 3: Table S3 for per-protocol analysis. Furthermore, Spearman’s correlation was used to assess the relationship between zonulin level and outcome.

### PRO got the effective descend on concentration of plasma endotoxin

**Figure 2:**
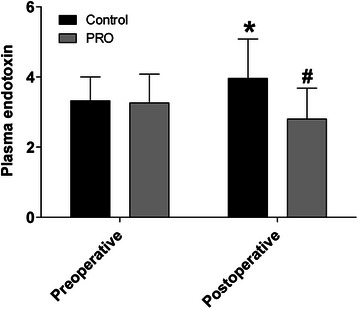
**Probiotics lowered the postoperative bacterial translocation and endotoxin (Intention-to-treat analysis).** PRO effectively decreased the plasma concentration of endotoxin in patients of colorectal liver metastases with normal postoperative intestinal barrier function, compared with the control group (n = 30 for control group and n = 30 for PRO group). Black bar represents the control group, and gray bar represents the PRO group. * (Control) vs. Preoperative, P < 0.05; ^#^ (PRO) vs. Preoperative, P < 0.05; * vs. ^#,^ P < 0.05. Numerical data are expressed as the means ± standard deviation, and compared by the t-test between groups. Plasma endotoxin was determined preoperatively (hospital admission day), and postoperatively (10 d treatment after surgery).

Patients with normal intestinal barrier function were further analyzed. Before treatment, the plasma concentrations of endotoxin indicated no significant difference (3.32 ± 0.68 in the control group vs. 3.26 ± 0.82 in the PRO group, P = 0.759). Control group showed a higher level of plasma endotoxin after 10 d postoperative treatment (3.96 ± 1.12 vs. 3.32 ± 0.68, P = 0.015), while PRO group showed a lower level of plasma endotoxin after treatment (2.80 ± 0.88 vs. 3.26 ± 0.82, P = 0.041). When the two groups were compared postoperatively, the PRO group was indicated significantly lower level of plasma endotoxin (3.96 ± 1.12 in the control group vs. 2.80 ± 0.88 in the PRO group, P < 0.001), compared with control group (Figure 2 & Additional file 5: Figure S1). Spearman’s correlation indicated that there was a direct correlation between the postoperative serum zonulin level and the plasma endotoxin (r = 0.962).

### PRO reduced postoperative infection rate and positive rate of the blood microbial DNA

To determine the types of infectious bacteria and positive rate of microbial culture, bacterial DNA levels in the blood, central line tips and sputum sample cultures were investigated. During the postoperative 72 h period, the growth rate of positive bacterial cultures (including blood, central lines and sputum) in the control group (53%, 16 in 30 patients) was significantly higher than the PRO group (23%, 7 in 30 patients), as shown in Table 3 (P =0.033). The positive rate of bacteria in the control group (30%, 9 in 30 patients) was also found significantly higher compared with the PRO group (7%, 2 in 30 patients, P = 0.042, Table 4 & Additional file 6: Table S5). Microbial DNA was found in all patients whose blood cultures were positive. The overall blood bacterial DNA positive rate in the control group (53%, 16/30) was significantly higher than in the PRO group (20%, 6/30, P = 0.015). Only 3 pathogens were detected in the blood samples, *Escherichia coli, Staphylococcus aureus* and *Aeruginosin*; *Escherichia coli* was the most common identified bacteria in 47.83% (11/23) of samples with bacteria.

**Table 4:** **Culture of bacterial culture of blood, central lines and sputum (Intention-to-treat)**

Sample	Control group (n = 30)			PRO group (n = 30)		
Bacterium	Blood	Central lines	sputum	Blood	Central lines	sputum
*Escherichia coli*	6	1	2	1	0	1
*Staphylococcus aureus*	2	1	2	1	1	1
*Klebsiella pneumoniae*	0	0	1	0	0	1
*Aeruginosin*	1	0	0	0	1	0
Bacterial positive patient	9	2	5	2	2	3
Total	16			7		

The total bacterial positive rate in control group was 53.33% (16 in 30 patients); in PRO group 23.33% (7 in 30 patients), P = 0.033; bacterial positive rate of the blood in the control group was 30.00% (9 in 30 patients), while in PRO group was 6.67% (2 in 30 patients), P = 0.042.

Nominal data by Pearson χ^2^ test or Fisher’s exact test between groups.

### PRO inhibit the p38 MAPK signaling pathway

It is reported that the p38 MAPK signaling pathway is involved in the protection of intestinal barrier function [27]. In this study, we further investigated the relation between p38 MAPK signaling pathway and the liver barrier function. To determine whether the PRO treatment was associated with the induction of p38 MAPK signaling pathway or not, samples from adjacent normal liver tissues (>1 cm form the metastatic tumor) were obtained from CLM patients (n = 5 for each group). The results showed that the expression of p38 MAPK was lower in the PRO group (1.26 ± 0.60), compared with the control group (2.28 ± 0.68, P = 0.033).

Randomized clinical trials regarding the effects of perioperative PRO treatment on the outcomes of colorectal cancer were rare, especially for CLM. Our pervious study indicated the perioperative use of PRO to protect human intestinal barrier function and prevent postoperative infectious complications after CRC surgery [1,11]. No study has reported the effects of pre and postoperative use of PRO on the liver barrier after CLM surgery. Here, we first investigated the relation between probiotics and the liver barrier clinically.

**Figure 3:**
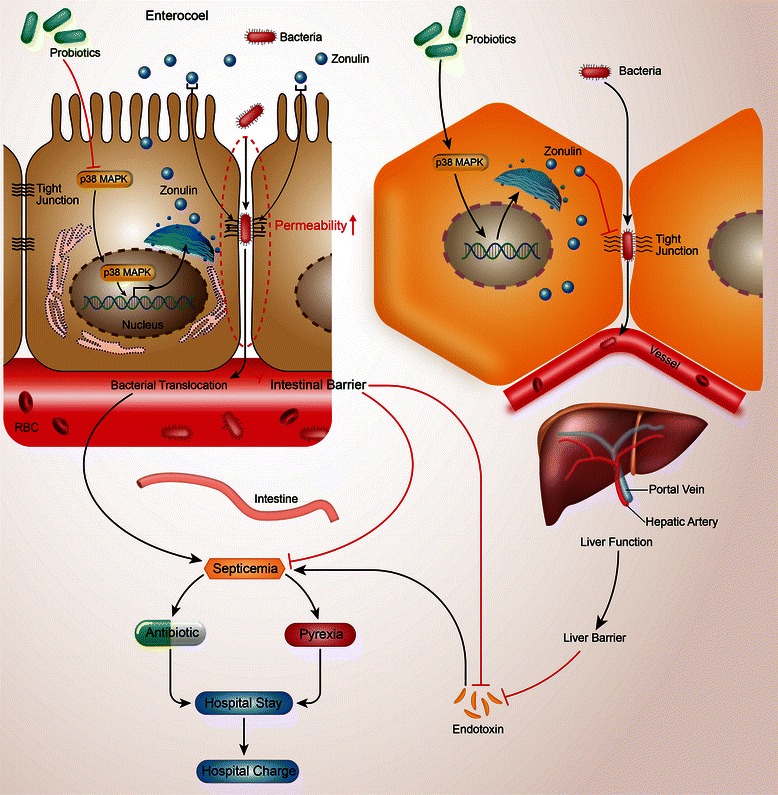
**Schematic diagram for the clinical regulatory pathway of probiotics.**

In our study, a double-center and double-blind randomized clinical trial was performed. Zonulin is a newly identified protein biomarker of barrier function [22,25,28]. Interestingly, we found the postoperative zonulin level (1.36 ± 0.50 ng/mg protein) of the control group was higher in CRC with liver metastases compared with that without liver metastases (1.08 ± 0.28 ng/mg protein) [1]. So we hypothesize liver barrier also contributed to the change of postoperative zonulin level in CLM. Results showed that PRO could efficiently lower the postoperative infection related complications in CLM patients with normal intestinal barrier function, which indicated that liver barrier dysfunction also contributed to the postoperative infection related complications of CLM patients. A novel concept of the “clinical regulatory pathway” (CP) was proposed [1], which was described as the mechanism by which a clinical treatment cause a series of sequenced molecular and clinical responses and just looks like the molecular signal transduction pathway. CP could link molecular signals and clinical outcomes more intuitively and vividly. So we drew a CP picture to elucidate the relation about PRO, liver barrier, intestinal barrier and postoperative infection related complications (Figure 3). We also promote the CP of the protective effects of PRO against postoperative infection related complications through the liver barrier in CLM patients: peri-operative administration of PRO might inhibit the p38 MAPK signaling pathway, which will extend the understanding of zonulin about its regulation on the barrier function after surgery [29-33].

Firstly, the serum zonulin level was not significantly different between the control and PRO groups, which was higher in the PRO group after 10 d postoperative treatment (Table 2). The incidence of postoperative septicemia was higher (87%, 26/30) in the control group compared with 50% (15/30) in the PRO group. However, either the septicemia rate was not shown significant difference between the two groups, while postoperative septicemia rate was significantly higher in the high serum zonulin group than the low serum zonulin group indicated septicemia may be regulated by the organ barrier. It shown that incidence of postoperative septicemia was correlated with the serum zonulin level (r = 0.613 for or the intention-to-treat analysis and r = 0.647 for per-protocol analysis). Therefore, we can deduce that PRO could efficiently reduce the zonulin level and then lower the incidence of postoperative septicemia (PRO → zonulin → septicemia).

Furthermore, duration of postoperative pyrexia, cumulative duration of antibiotic therapy and postoperative hospital stay were also suggesting the regulation by zonulin. Combined with the, we can deduce that: PRO → zonulin → septicemia → duration of postoperative pyrexia time → duration of antibiotic therapy → postoperative hospital stay → hospital charge.

The finding that *Escherichia coli* was the main bacteria identified in blood samples (Table 3) indicated that pathogen translocated from the intestinal tract to the blood through the liver barrier is the most important reason for postoperative septicemia in patients without intestinal barrier dysfunction [1,11]. We deduce that perioperative use of PRO may reduce postoperative zonulin levels, injure the liver barrier and so on. (PRO → zonulin → liver barrier → septicemia → duration of postoperative pyrexia time → duration of antibiotic therapy → postoperative hospital stay → hospital charge).

Combined with the overall liver function indexes (Table 2), both ALT and AST were significantly lowered by the treatment of PRO, indicating the protection of PRO on liver function, which may contribute to the injury of liver function [34]. Liver function can be added to the CP pathway: PRO → zonulin → liver function → liver barrier → septicemia → duration of postoperative pyrexia time → duration of antibiotic therapy → postoperative hospital stay → hospital charge.

Investigation of the plasma endotoxin indicated no significant difference before treatment (P = 0.759). Control group showed a higher level, whereas PRO group showed a lower level of plasma endotoxin after 10 d postoperative treatment, which showed a significant difference. Spearman’s correlation indicated a direct correlation between the postoperative serum zonulin level and the plasma endotoxin (r = 0.962). As is well-known that septicemia can be caused by endotoxin [35], we further improve our CP pathway as: PRO → zonulin → liver function → liver barrier → endotoxin → septicemia → duration of postoperative pyrexia time → duration of antibiotic therapy → postoperative hospital stay → hospital charge.

P38 MAPK, a Ser/Thr kinase belonging to the family of MAPKs, was selected as the signal molecular in the regulation of zonlulin, which is related to the expression level of several inflammatory genes, while inhibition of p38 MAPK phosphorylation by PRO could protect intestinal barrier from dysfunction [27]. Our prevoious study also showed that PRO could inhibit the expression of p38 MAPK, lower the clinical effects of zonulin and decrease the intestinal permeability [1]. Accordingly, we hypothesize that the effects of PRO were mediated via the p38 MAPK pathway and then play its role in the liver barrier. Results verified that the expression level of p38 MAPK was lower in the PRO group compared with the control group (P = 0.033). Above all, our CP of PRO on postoperative septicemia in colorectal cancer surgery can be presented as: PRO → p38 MAPK → zonulin → liver function → liver barrier → endotoxin → septicemia → duration of postoperative pyrexia time → duration of antibiotic therapy → postoperative hospital stay → hospital charge. Combined with the CP that PRO regulate intestinal barrier, we deduce that PRO regulate postoperative infection related complications in patients of CLM via two pathway—intestinal barrier and liver barrier (Figure 3).

It is reported that for severe acute pancreatitis patients, prophylactic use of PRO could not only fail to reduce the risk of occurrence of infectious complications, but, on the contrary, increase the patients’ mortality due to the high oxygen demand and the severe gastrointestinal ischemia [36]. While in our study, no death case was reported, which may because the intestinal barrier and liver barrier was not injured so seriously. One of the advantages is that although our study is a double → centered one, the interference of different operators to the study results was not the most important [37].

We only pointed out one of the possible signal transduction pathways about the zonulin expression regulated by PRO [38]. It is quite reasonable that zonulin may in turn have a regulation on p38 MAPK pathway or a double regulation between P38 MAPK and zonulin, given similar signaling of the zonulin prokariotic analogue Zot [39],further basic studies may be needed Assessment of numerous outcome gives rise to a multiple comparison issue, especially marginally significant results, which require cautious analyse. Fortunately, the consistency of findings lends support to the effectiveness of treatment with PRO [11]. Comparison of HZ and LZ subsets suffered from small sample size and not randomized comparison issues and the randomization protection also does not apply.

## Conclusion

To sum up, perioperative PRO treatment could reduce the rate of postoperative septicemia and maintain the liver barrier in patients undergoing CLM surgery, which is associated with reduced serum zonulin level. We propose a new model about the regulation of PRO to liver barrier via CP (Figure 3). Serum zonulin levels could be an early biomarker for septicemia. We recommend the preoperative oral intake of PRO combined with postoperative PRO treatment in patients who underwent CLM surgery.

## Additional files

Additional file 1: Table S1. Baseline of characteristics of the patients with colorectal liver metastases undergoing surgery at hospital admission in the study (Per-protocol). Additional file 2: Table S2. Comparison of postoperative outcomes between probiotics and control (Per-protocol). Additional file 3: Table S3. Comparison of serum zonulin with the postoperative infectious complications between probiotics and control the patients with normal intestinal barrier function (Per-protocol). Additional file 4: Table S4. Baseline of characteristics of the patients with normal intestinal barrier function. Additional file 5: Figure S1. PRO lowered the postoperative bacterial translocation and endotoxin (Per-protocol analysis). PRO effectively decreased the plasma concentration of endotoxin in patients of colorectal liver metastases with normal postoperative intestinal barrier function, compared with the control group (n = 28 for control group and n = 29 for PRO group). Black bar represents the control group, and gray bar represents the PRO group. * (Control) vs. Preoperative, P < 0.05; # (PRO) vs. Preoperative, P < 0.05; * vs. #, P < 0.05. Numerical data are expressed as the means ± standard deviation, and compared by the t-test between groups. Plasma endotoxin was determined preoperatively (hospital admission day), and postoperatively (10 d treatment after surgery). Additional file 6: Table S5. Culture of bacterial culture of blood, central lines and sputum (Per-protocol).

## Abbreviations

CRC: Colorectal cancer
CLM: Colorectal liver metastases
ALT: Alanine transarninase
AST: Aspartate aminotransferase
Zot: Zonula occludens toxin
BL: *Bifido-bacterium longum*
BMI: Body mass index
CP: Clinical regulatory pathway
ELISA: Enzyme-linked immunosorbent assay
Hb: Hemoglobin
LA: *Lactobacillus acidophilus*
LP: *Lactobacillus plantarum*
L/M: Lactulose/mannitol
IL: Interleukin
PRO: Probiotics
TBS-T: Tris-buffered saline 0.05% Tween 20
