# Supplementary material for: Positive regulatory effects of perioperative probiotic treatment on postoperative liver complications after colorectal liver metastases surgery: a double-center and double-blind randomized clinical trial
Source: BMC Gastroenterol. 2015 Mar 20;15:34. doi: 10.1186/s12876-015-0260-z (PMC4374379; doi:10.1186/s12876-015-0260-z)
Supplement: Additional file 1: Table S1. — Baseline of characteristics of the patients with colorectal liver metastases undergoing surgery at hospital admission in the study (Per-protocol). [file 12876_2015_260_MOESM1_ESM.zip › 12876_2015_260_add1.rtf]

Table S1. Baseline of characteristics of the patients with colorectal liver metastases undergoing surgery at hospital admission in the study (Per-protocol)

Index	Control group (n=58)	PRO group (n=59)	
Sex (Male/Female)	30/28	31/28	
Age (Year)	61.22±16.02	63.98±18.08	
BMI (kg/m2)	23.12±5.20	22.32±3.62	
Location of tumor			
ascending colon	12	15	
Transverse colon	5	5	
Descending colon	11	11	
Sigmoid colon	12	10	
Rectum 	18	18	
Time between onset of symptoms and hospital admission (d)	50.66±16.26	55.06±18.12	
Preoperative albumin (g/dL)	38.02±8.28	36.92±6.82	
Preoperative Hb (g/L)	122.08±31.26	116.68±35.50	
Creatinine (mg/dL)	1.28±0.26	1.22±0.66	
Operative time (min)		180.60±52.02		192.06±50.02	
Intra-operative blood loss (ml)	352.12±188.06	358.22±160.08	
Transfusion during operation (ml)	326.10±126.06	323.22±136.92	
Usage of supplemental albumin postoperation (g)	21.22±16.28	26.28±20.02	
Preoperation prepared time (d)	6.68±3.56	6.60±2.90	
Metronidazole (n)	58	59	
Penicillin (n)	22	29	
Ceftriaxone (n)	36	30	
ALT (U/L)	32.08±15.52	31.86±18.28	
AST (U/L)	28.02±18.26	29.20±19.22	

BMI, body mass index; Hb, hemoglobin; ALT, alanine transarninase (normal value, 0-40 U/L); AST, aspartate aminotransferase (normal value, 0-40 U/L);
There were no significant differences about the characteristics between the two groups;
Quantitative data are expressed as mean ± standard deviation. Numerical data were compared by t test and nominal data by Pearson ÷2 test or Fisher's exact test between groups.
