# Supplementary material for: Positive regulatory effects of perioperative probiotic treatment on postoperative liver complications after colorectal liver metastases surgery: a double-center and double-blind randomized clinical trial
Source: BMC Gastroenterol. 2015 Mar 20;15:34. doi: 10.1186/s12876-015-0260-z (PMC4374379; doi:10.1186/s12876-015-0260-z)
Supplement: Additional file 2: Table S2. — Comparison of postoperative outcomes between probiotics and control (Per-protocol). [file 12876_2015_260_MOESM2_ESM.zip › 12876_2015_260_add2.rtf]

Table S2 Comparison of postoperative outcomes between probiotics and control (Per-protocol)

Outcomes	Per-protocol	
	Control (n = 58)	PRO (n = 59)	P Value	
Septicemia incidence (%)	86 (50/58)	59 (35/59)	0.002	
ALT (U/L)	56.96±18.28	38.56±19.98	<0.001	
AST (U/L)	46.26±23.12	35.58±20.96	0.010	
Intro-abdominal drainage time (d)	4.3±1.8	4.6±1.6	0.342	
Incision infection (%)	14 (8/58)	8 (5/59)	0.394	
Central lines infection (%)	7 (4/58)	3 (2/59)	0.439	
Pneumonia infection (%)	10 (6/58)	2 (1/59)	0.061	
Urinary infection (%)	12 (7/58)	2 (1/59)	0.032	
First defecation time (d)	3.5±1.8	2.6±1.8	0.008	
Diarrhea incidence£¨%£©	46 (26/58)	24 (15/59)	0.034	
Urinary catheters time (d)	6.8±2.6	6.5±2.2	0.549	
Abdominal cramping (%)	50 (29/58)	20 (12/59)	<0.001	
Abdominal distension (%)	52 (30/58)	31 (18/59)	0.025	
Intake time of fluid diet (d)	3.5±1.2	3.0±1.6	0.059	
Intake time of solid diet (d)	5.6±1.8	5.0±1.8	0.074	
Side effects of probiotic use	0	0	N/A	
Duration of postoperative pyrexia (>38.5 °C) (d)	6.96±2.20	6.10±1.66	0.018	
Hypoalbuminemia (%)	45 (26/58)	27 (16/59)	0.035	
Cumulative duration of antibiotic therapy	7.62±2.16	6.28±2.02	<0.001	
Postoperative hospital stay	12.28±3.12	11.06±2.62	0.024	
Hospital charge (Yuan)	58696.08 ± 11286.36	51698.82 ± 11386.12	0.001	
Death case	0	0	N/A	
Serum zonulin (ng/mg protein)	1.38 ± 0.52	0.39 ± 0.38	<0.001	
ALT, alanine transarninase (normal value, 0-40 U/L); AST, aspartate aminotransferase (normal value, 0-40 U/L);
