# Supplementary material for: Positive regulatory effects of perioperative probiotic treatment on postoperative liver complications after colorectal liver metastases surgery: a double-center and double-blind randomized clinical trial
Source: BMC Gastroenterol. 2015 Mar 20;15:34. doi: 10.1186/s12876-015-0260-z (PMC4374379; doi:10.1186/s12876-015-0260-z)
Supplement: Additional file 3: Table S3. — Comparison of serum zonulin with the postoperative infectious complications between probiotics and control the patients with normal intestinal barrier function (Per-protocol). [file 12876_2015_260_MOESM3_ESM.zip › 12876_2015_260_add3.rtf]

Table S3 Comparison of serum zonulin with the postoperative infectious complications between probiotics and control the patients with normal intestinal barrier function (Per-protocol)

Outcomes	Per-protocol	
	Control (n = 28)	PRO (n = 29)	P Value	
Serum zonulin (ng/mg protein)	0.75 ± 0.26	0.51 ± 0.29	0.002	
Septicemia (%)							
Total	89 (25/28)	52 (15/29)	0.003	
HZ	95 (19/20)	71 (10/14)	0.136	
LZ	63 (5/8)	33 (5/15)	0.221	
HZ vs. LZ	HZ vs LZ (85% vs 43%), P<0.001	
Correlation between septicemia and zonulin, r = 0.647, P<0.001	
Urinary infection (%)							
Total	18 (5/28)	0 (0/29)	0.023	
HZ	15 (3/20)	0 (0/14)	0.251	
LZ	25 (2/8)	0 (0/15)	0.111	
HZ vs. LZ	HZ vs LZ (8% vs 9%), P=1.000	
Diarrhea incidence (%)							
Total	61 (17/28)	28 (8/29)	0.017	
HZ	50 (10/20)	29 (4/14)	0.296	
LZ	88 (7/8)	27 (4/15)	0.009	
HZ vs. LZ	HZ vs LZ (41% vs 48%), P=0.786	
Duration of postoperative pyrexia (>38.5°C) (d)							
Total	7.16 ± 2.33	5.41 ± 3.23	0.023	
HZ	7.97 ± 1.77 (n=20)	8.04 ± 0.90 (n=14)	0.904	
LZ	5.14 ± 2.43 (n=8)	2.95 ± 2.61 (n=15)	0.063	
HZ vs. LZ	HZ vs LZ (8.00± 1.46 vs 3.71 ± 2.71), P<0.001	
Cumulative duration of antibiotic therapy (d)							
Total	7.25±1.62	6.10±1.74	0.013	
HZ	7.90 ± 1.21 (n=20)	7.50 ± 1.22 (n=14)	0.352	
LZ	5.63 ± 1.41 (n=8)	4.80 ± 0.94 (n=15)	0.107	
HZ vs. LZ	HZ vs LZ (7.74± 1.21 vs 5.09 ± 1.16), P<0.001	
Postoperative hospital stay				
Total	13.00±3.08	11.31±2.25	0.021	
HZ	14.15 ± 2.64 (n=20)	13.21 ± 1.63 (n=14)	0.248	
LZ	10.13 ± 2.10 (n=8)	9.53 ± 0.83 (n=15)	0.342	
HZ vs. LZ	HZ vs LZ (13.76± 2.30 vs 9.74 ± 1.39), P<0.001	
Hospital charge (Yuan)	60186.36 ± 6568.28	53668.26 ± 6526.86	<0.001	
HZ, high serum zonulin (≥0.6 ng/mg protein); LZ, low serum zonulin (<0.6 ng/mg protein); total = HZ+LZ; NS, No significance.
Numerical data between groups were compared by the t-test and nominal data by Pearson ÷2 test or Fisher's exact test.
HZ vs LZ compares these subgroups without regard to treatment.
There was also a significant correlation between zonulin and duration of postoperative pyrexia (r = 0.920, p<0.001), cumulative duration of antibiotic therapy and zonulin (r = 0.935, p<0.001), and postoperative hospital stay (r = 0.909, p<0.001).
