# Supplementary material for: Positive regulatory effects of perioperative probiotic treatment on postoperative liver complications after colorectal liver metastases surgery: a double-center and double-blind randomized clinical trial
Source: BMC Gastroenterol. 2015 Mar 20;15:34. doi: 10.1186/s12876-015-0260-z (PMC4374379; doi:10.1186/s12876-015-0260-z)
Supplement: Additional file 4: Table S4. — Baseline of characteristics of the patients with normal intestinal barrier function. [file 12876_2015_260_MOESM4_ESM.zip › 12876_2015_260_add4.rtf]

Table S4. Baseline of characteristics of the patients with normal intestinal barrier function

Index	Intention-to-treat	Per-protocol	
	Control group (n=30)	PRO group (n=30)	Control group (n=26)	PRO group (n=28)	
Sex (Male/Female)	16/14	17/13	14/12	16/12	
Age (Year)	62.20±16.18	66.08±19.22	62.62±16.96	65.88±18.68	
BMI (kg/m2)	22.82±5.02	22.16±3.98	23.06±5.26	22.39±3.82	
Location of tumor					
ascending colon	5	6	5	5	
Transverse colon	3	3	2	3	
Descending colon	6	6	5	6	
Sigmoid colon	6	5	5	5	
Rectum 	10	10	9	9	
Time between onset of symptoms and hospital admission (d)	52.06±15.02	53.26±16.20	51.26±15.22	53.22±16.28	
Preoperative albumin (g/dL)	36.22±6.50	36.55±5.12	36.06±6.56	36.56±5.32	
Preoperative Hb (g/L)	120.12±26.56	112.28±30.58	120.58±28.50	115.62±31.02	
Creatinine (mg/dL)	1.28±0.18	1.22±0.56	1.26±0.16	1.22±0.52	
Operative time (min)	192.66±55.88	198.92±55.02	191.26±56.06	199.02±56.02	
Intra-operative blood loss (ml)	312.06±168.96	350.68±160.18	316.02±160.52	352.16±166.22	
Transfusion during operation (ml)	311.22±126.32	336.12±128.60	312.10±126.86	335.28±129.96	
Usage of supplemental albumin postoperation (g)	21.28±12.38	26.26±19.28	21.26±12.20	26.18±18.96	
Preoperation prepared time (d)	6.26±3.08	6.66±2.16	6.30±3.12	6.65±2.26	
Metronidazole (n)	30	30	26	28	
Penicillin (n)	12	15	11	14	
Ceftriaxone (n)	18	15	15	14	
ALT (U/L)	32.28±10.96	33.10±15.26	32.18±10.56	33.82±15.22	
AST (U/L)	26.66±12.18	25.36±15.28	26.02±12.22	25.28±15.36	

BMI, body mass index; Hb, hemoglobin; ALT, alanine transarninase (normal value, 0-40 U/L); AST, aspartate aminotransferase (normal value, 0-40 U/L);
There were no significant differences about the characteristics between the two groups both in the intention-to-treat analysis and per-protocol analysis;
Quantitative data are expressed as mean ± standard deviation. Numerical data were compared by t test and nominal data by Pearson ÷2 test or Fisher's exact test between groups.
