# Supplementary figures and images for: Positive regulatory effects of perioperative probiotic treatment on postoperative liver complications after colorectal liver metastases surgery: a double-center and double-blind randomized clinical trial
Source: BMC Gastroenterol. 2015 Mar 20;15:34. doi: 10.1186/s12876-015-0260-z (PMC4374379; doi:10.1186/s12876-015-0260-z)

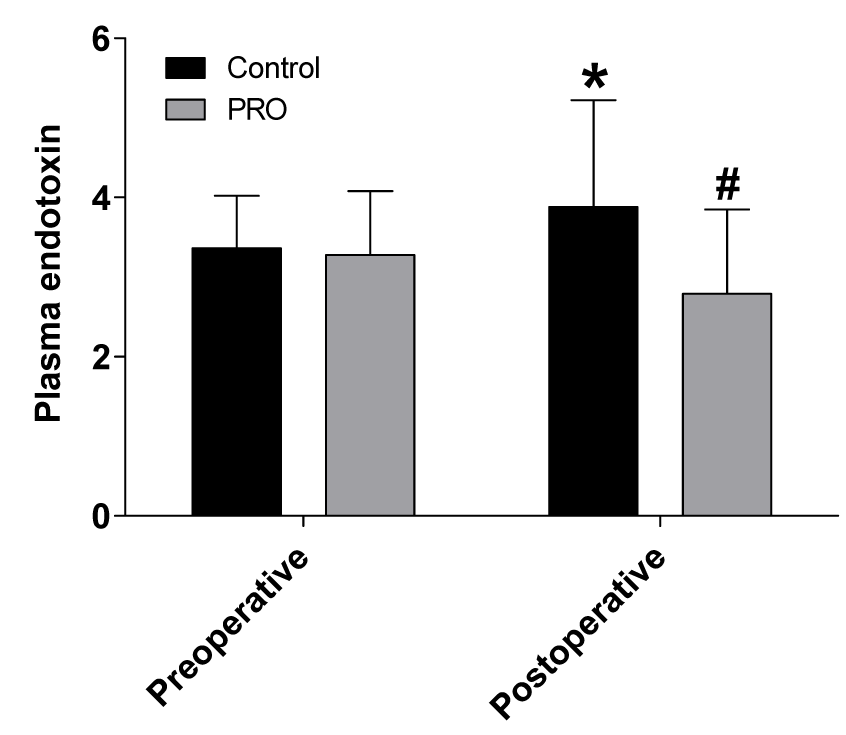

Supplement: Additional file 5: Figure S1. — PRO lowered the postoperative bacterial translocation and endotoxin (Per-protocol analysis). PRO effectively decreased the plasma concentration of endotoxin in patients of colorectal liver metastases with normal postoperative intestinal barrier function, compared with the control group (n = 28 for control group and n = 29 for PRO group). Black bar represents the control group, and gray bar represents the PRO group. * (Control) vs. Preoperative, P < 0.05; # (PRO) vs. Preoperative, P < 0.05; * vs. #, P < 0.05. Numerical data are expressed as the means ± standard deviation, and compared by the t-test between groups. Plasma endotoxin was determined preoperatively (hospital admission day), and postoperatively (10 d treatment after surgery). [file 12876_2015_260_MOESM5_ESM.tiff]
