# Supplementary material for: Positive regulatory effects of perioperative probiotic treatment on postoperative liver complications after colorectal liver metastases surgery: a double-center and double-blind randomized clinical trial
Source: BMC Gastroenterol. 2015 Mar 20;15:34. doi: 10.1186/s12876-015-0260-z (PMC4374379; doi:10.1186/s12876-015-0260-z)
Supplement: Additional file 6: Table S5. — Culture of bacterial culture of blood, central lines and sputum (Per-protocol). [file 12876_2015_260_MOESM6_ESM.zip › 12876_2015_260_add6.rtf]

Table S5 Culture of bacterial culture of blood, central lines and sputum (Per-protocol)

Sample
Bacterium	Control group (n=28)	PRO group (n=29)	
	Blood	Central lines	sputum	Blood	Central lines	sputum	
Escherichia coli	5	1	2	1	0	1	
Staphylococcus aureus	2	1	2	1	1	1	
Klebsiella pneumoniae	0	0	1	0	0	1	
Aeruginosin	1	0	0	0	1	0	
Bacterial positive patient	8	2	5	2	2	3	
Total	15	7	
The total bacterial positive rate in control group was 53.57.00% (15 in 28 patients); in PRO group 24.14% (7 in 29 patients), P=0.031; bacterial positive rate of the blood in the control group was 28.57% (8 in 28 patients), while in PRO group was 6.70% (2 in 29 patients), P=0.041;
Nominal data by Pearson ÷2 test or Fisher's exact test between groups. 
